# 3D-printed individualized tooth-borne tissue retraction devices compared to conventional dental splints for head and neck cancer radiotherapy: a randomized controlled trial

**DOI:** 10.1186/s13014-021-01803-8

**Published:** 2021-04-17

**Authors:** Thomas Held, Christopher Herpel, Franz Sebastian Schwindling, Leo Christ, Kristin Lang, Sebastian Regnery, Tanja Eichkorn, Adriane Hommertgen, Cornelia Jaekel, Johannes Krisam, Julius Moratin, Jan Mrosek, Karl Metzger, Karim Zaoui, Tracy Moutsis, Semi Harrabi, Klaus Herfarth, Christian Freudlsperger, Peter Rammelsberg, Jürgen Debus, Sebastian Adeberg

**Affiliations:** 1 Department of Radiation Oncology, Heidelberg University Hospital, Im Neuenheimer Feld 400, 69120 Heidelberg, Germany; 2 Heidelberg Institute of Radiation Oncology (HIRO), Heidelberg, Germany; 3 National Center for Tumor Diseases (NCT), Heidelberg, Germany; 4 Clinical Cooperation Unit Radiation Oncology, German Cancer Research Center (DKFZ), Heidelberg, Germany; 5 Heidelberg Ion-Beam Therapy Center (HIT), Heidelberg, Germany; 6 German Cancer Consortium (DKTK), partner site Heidelberg, German Cancer Research Center (DKFZ), Heidelberg, Germany; 7 Department of Prosthetic Dentistry, Heidelberg University Hospital, Heidelberg, Germany; 8 Institute of Medical Biometry and Informatics (IMBI), Heidelberg University Hospital, Heidelberg, Germany; 9 Department of Oral and Maxillofacial Surgery, University Hospital Heidelberg, Heidelberg, Germany; 10 Department of Otorhinolaryngology, University of Heidelberg, Heidelberg, Germany

**Keywords:** Intensity-modulated radiotherapy, Particle therapy, Toxicity, Radiation-induced oral mucositis, Xerostomia, Squamous cell carcinoma, Salivary gland tumor

## Abstract

**Background:**

Despite modern treatment techniques, radiotherapy (RT) in patients with head and neck cancer (HNC) may be associated with high rates of acute and late treatment-related toxicity. The most effective approach to reduce sequelae after RT is to avoid as best as possible healthy tissues and organs at risk from the radiation target volume. Even small geometric changes can lead to a significant dose reduction in normal tissue and better treatment tolerability. The major objective of the current study is to investigate 3D printed, tooth-borne tissue retraction devices (TRDs) compared to conventional dental splints for head and neck RT.

**Methods:**

In the current two-arm randomized controlled phase II trial, a maximum of 34 patients with HNC will be enrolled. Patients will receive either TRDs or conventional dental splints (randomization ratio 1:1) for the RT. The definition of the target volume, modality, total dose, fractionation, and imaging guidance is not study-specific. The primary endpoint of the study is the rate of acute radiation-induced oral mucositis after RT. The quality of life, local control and overall survival 12 months after RT are the secondary endpoints. Also, patient-reported outcomes and dental status, as well as RT plan comparisons and robustness analyzes, will be assessed as exploratory endpoints. Finally, mesenchymal stem cells, derived from the patients’ gingiva, will be tested in vitro for regenerative and radioprotective properties.

**Discussion:**

The preliminary clinical application of TRD showed a high potential for reducing acute and late toxicity of RT in patients with HNC. The current randomized study is the first to prospectively investigate the clinical tolerability and efficacy of TRDs for radiation treatment of head and neck tumors.

*Trial registration*: ClinicalTrials.gov; NCT04454697; July 1^st^ 2020; https://clinicaltrials.gov/ct2/show/record/NCT04454697.

## Background

Despite modern radiation techniques, e.g. intensity-modulated radiotherapy (IMRT), radiation treatment may be associated with high rates of severe treatment-related toxicity [1, 2]. Higher grade radiation-induced oral mucositis (RIOM) (≥ Common Terminology Criterion [CTC] grade III), common dose-limiting toxicity, occurs in up to 60% of patients [3–5]. The rate of RIOM is further increased by the application of simultaneous systemic therapies (e.g., chemotherapy). The immediate consequences of this are limited tolerability of therapy and reduced compliance, and an interruption or even discontiuation of treatment [6]. If epitheliolysis and ulcerations occur, the risk of bacterial superinfection with possible complications, including sepsis, increases. In the long term, mucositis causes dry mouth, taste disorders, pain, and difficulty swallowing, and weight loss, which increases the frequency of hospitalization. Many prospective clinical studies demonstrated a reduction in the quality of life in this context [7, 8].

The probability of radiogenic damage to epithelial cells is dose-dependent [9]. Established risk factors for the development of oral mucositis during radiotherapy (RT) include decreased oral hygiene and smoking. So far, many approaches have been postulated for the prevention and treatment of mucositis, but there are no clear recommendations other than adequate care and analgesia. A total of 320 studies listed about oral mucositis at "http://www.clinicaltrials.gov" underscore the clinical relevance of the topic.

However, the most effective approach to reducing acute toxicity after RT is to avoid as best as possible healthy tissues and organs at risk from the high-dose area of the radiation field. Isolated case reports [10] and several retrospective studies [11–13] on customized TRDs for RT of head and neck tumors reported reduced rates of mucositis with good tolerability. Currently, there are no prospective studies available.

Usually, there is a close positional relationship in the head and neck area between the gross tumor volume and the adjacent normal tissue and organs at risk. Due to anatomical reasons, including parts of the tongue, jaw, or oral mucosa in the high-dose RT area is often unavoidable. Using TRDs can increase the distance between the tumor and normal tissue in certain areas. Even the smallest geometric change of a few millimeters can significantly reduce the dose of normal tissue with significant benefits for the patient [14]. This is essential in particular for high precision RT, e.g. by IMRT, MRI-guided RT, or heavy ion therapy. Another factor of uncertainty is the mobility of the tongue and the base of the tongue because there is usually no fixation during RT. Proper immobilization can reduce unwanted movements and allow for more precise therapy. Besides increasing the distance between the tumor and healthy tissue to reduce RIOM, TRDs could also be advantageous for precise repositioning of the patient.

Traditionally, manufacturing TRDs that target healthy tissue displacement, tongue immobilization, and precise patient repositioning is complex and time-consuming [15]. This could be the reason conventional dental splints, made of thermoplastic resin, are used as the standard of clinical treatment in many institutions. Therefore, these thermoplastic dental splints will be used in the control arm of the present study. Introducing novel computer-assisted design and manufacturing techniques, e.g. 3D printing, has the potential to simplify TRD's workflow while reducing costs and increasing the quality of TRD. For certain indications, e.g. craniomandibular disorders, the production of aligners and TRDs using the 3D printing process is already established in our institution [16, 17]. The materials and methods used are approved and tested for intraoral use. The 3D printing process enables economical and flexible production.

The current trial aims to evaluate TRDs for individualized and effective RT of patients with malignant HNC to protect normal tissue and improve treatment tolerability. At the cellular level, the high regenerative potential of mesenchymal stem cells (MSCs) has been characterized in detail [18]. They can be isolated from various tissues such as bone marrow and adipose tissue, but also the head and neck region (including the gingiva). The importance of gingival MSCs for radioprotection in the head and neck area has not been elucidated.

## Methods/design

**Fig. 1:**
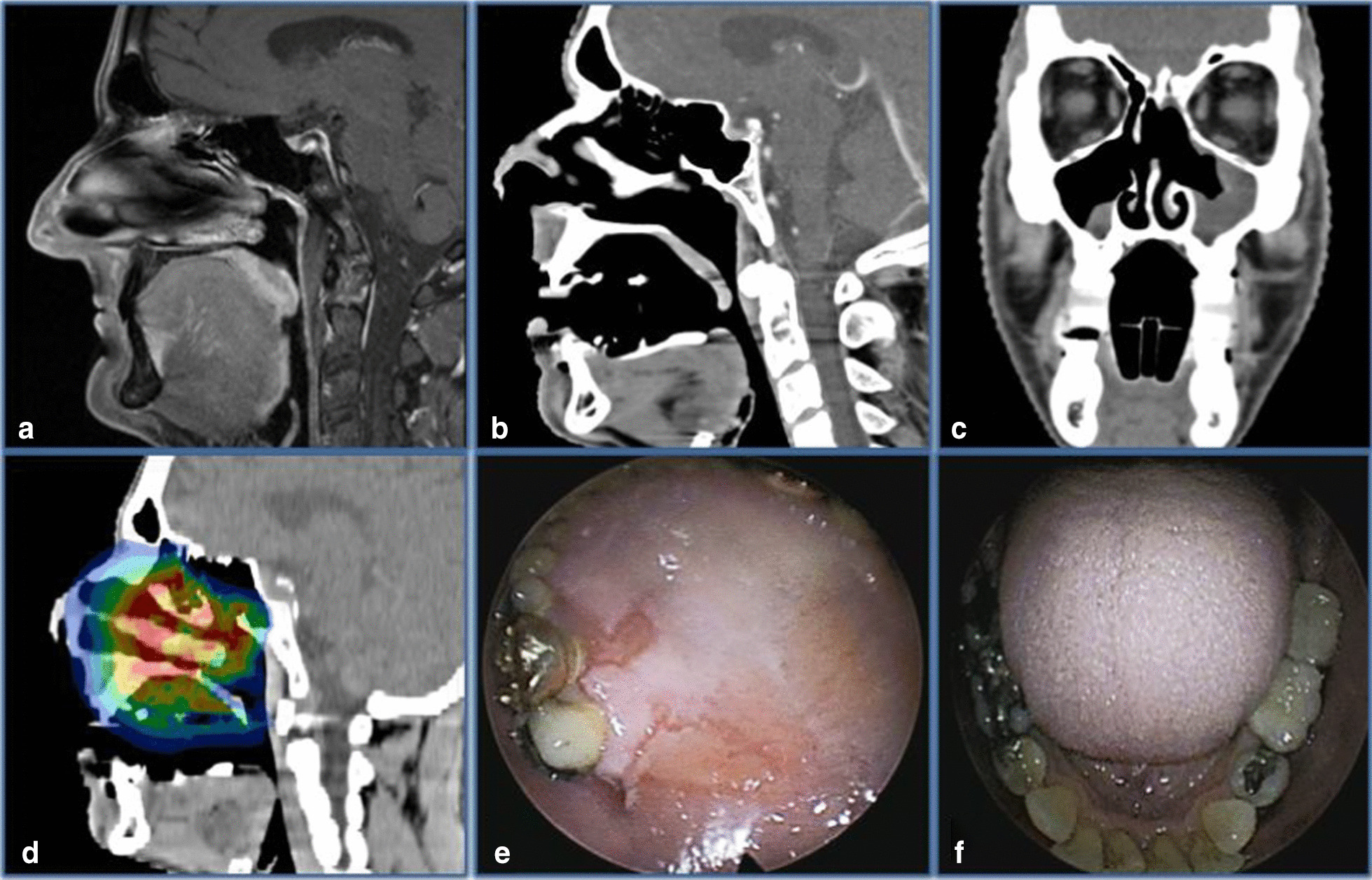
**a** T1 contrast-enhanced fat-saturated magnetic resonance imaging of a patient with sarcoma of the paranasal sinuses without tissue retraction device (TRD). **b**, **c** Contrast-enhanced computed tomography of the same patient with TRD. **d** Radiation treatment plan with TRD in situ. **e** Oral mucositis CTC grade III in the high-dose area of the radiation field. **f** Nonirritated tongue surface directly opposite the high-dose area of the radiation field

The current single-center, two-arm, randomized controlled phase II trial anticipates an enrollment of a maximum of 34 patients who meet the inclusion criteria. Patients will receive 3D printed dental TRDs or conventional dental splints (in a randomization ratio of 1:1 via block randomization using an online randomization tool). TRDs are pre-fabricated using 3D printing and are tailored to the individual patient and treatment intention. Each TRD comprises a fixation piece on the tooth which allows the opening of the mouth and the mandibular protrusion, the reproducible positioning, and the covering of the teeth in combination with lip- and cheek spacing. Also, a retraction part allows the displacement of the tongue. An example of a clinical case is shown in Fig. 1. A publication summarizing details of the design process and TRD fabrication is currently under preparation. Conventional dental splints are protective plastic stents covering the teeth to reduce the radiation overdose caused by dental restoration materials. No additional retraction parts or spacing devices are attached.

About 50–70% of patients require tooth extractions, e.g. due to caries, before RT [19]. In general, small amounts of periodontal tissue remain attached to the surface of the extracted teeth. From these tissue probes, isolation and expansion of the gingival MSCs will be performed. After isolation and expansion, clonogenicity and proliferation are examined in cell culture before and after in vitro radiation. Also, functional properties such as cell adhesion and migration are characterized, and the capacity for differentiation. Regenerative and radioprotective factors eventually correlate with the systematically recorded toxicity profile of patients.

To evaluate the consequences of TRDs on the integral dose to adjacent normal tissue, plan comparisons will be made between all patients in both treatment groups. In selected patients with available pre-treatment diagnostic computed tomography (CT) in the experimental arm, in silico intra-patient plan comparisons will further clarify the potential clinical benefits of TRDs concerning dose distribution. The robustness of TRD treatment plans concerning positional variability will be assessed as part of the routine daily imaging guide based on cone beam CT or orthogonal X-rays during treatment. The mean displacement of osseous reference points in the upper and lower jaw will be compared between both groups to assess the maxillomandibular position changes during RT. The total duration of the study is scheduled to be 36 months, including a 24-month recruitment phase and a minimum follow-up of 12 months.

### Inclusion criteria

The inclusion criteria are: diagnosed malignant tumors in the head and neck region; clinical target volume includes parts of one or several of the following anatomical regions: the upper jaw, the lower jaw, the hard palate, the tongue, the floor of the mouth, the buccal mucosa, the soft palate and the base of the tongue; an indication of adjuvant or definitive RT; age ≥ 18 years; Karnofsky performance score ≥ 60; complete wound healing after the surgical intervention; written informed consent; the ability of the subject to understand the character and individual consequences of the trial; for women of childbearing age (and men), adequate contraception.

### Exclusion criteria

Exclusion criteria are: previous head and neck RT; multifocal diffuse tumor growth; patients who have not recovered from the acute toxicities of previous therapies; trismus (mouth opening ≤ 2 cm); simultaneous chemo/immunotherapy; evidence that the patient cannot adhere to the study protocol (e.g., non-compliance); the refusal of patients to participate in the study.

### Radiation therapy

Radiation treatment will be carried out according to the current standard in our institution, following current clinical guidelines for HNC. The definition of the target volume, modality, total dose, fractionation, and imaging guidance is not study-specific. Simultaneous systemic therapies, particularly chemo- or immunotherapy, are not allowed in the current study. Due to the various histological entities projected for the current trial, a wide range of different treatment approaches is possible.

For treatment planning, patients will be immobilized with an individual immobilization mask. All patients will receive a non-contrast planning CT scan with a layer thickness of 3 mm and, if possible, also a contrast-enhanced CT or magnetic resonance imaging (MRI) for an optimal definition of the target volume. Treatment planning will be carried out using Syngo PT-Planning (Siemens, Erlangen, Germany) and RayStation® (RaySearch Laboratories, Stockholm, Sweden) planning software. Automated multi-atlas-based segmentation of normal tissue and organs at risk, including salivary glands, tongue, hard and soft palate, and mandible, will be performed according to EORTC guidelines using the software RayStation®. Dose constraints for normal tissues and organs will be respected according to the Quantitative Analyses of Normal Tissue Effects in the Clinic (QUANTEC) [20, 21]. According to standard procedures in our clinic, IMRT will be performed 5 fractions per week under image guidance with daily CT images and position correction using volumetric modulated arc therapy. If indicated, particle therapy will be done 5–6 fractions per week with protons or carbon ions. The active raster scanning technology will be used for the application of orthogonal X-ray image guidance and daily position correction. The target volume definition and the dose prescription will be left to the discretion of the treating radiation oncologist, following current clinical guidelines for HNC [22, 23].

### Study endpoints

The primary endpoint of the study is the rate of acute RIOM after RT with TRD compared to conventional dental splints. To allow an objective evaluation of RIOM complementary to clinical questionnaires, the oral cavity and oropharynx are subdivided into eight anatomical regions (Table 1). Based on previous RIOM studies [3–5], we defined the assumed rate of severe oral mucositis (≥ CTC grade III) as 60% in the treated anatomical region and its direct proximity. The primary target value is to prevent severe RIOM (≥ CTC grade III) in the anatomical regions directly adjacent to the CTV in 75% of patients with TRD. To assess the primary endpoint, all patients receive a diagnostic video endoscopy of the oral cavity and oropharynx performed by an ENT specialist directly after the last radiation treatment. A blinded evaluation of the mucositis grade will be performed.

**Table 1:** Anatomical regions of the oral cavity and oropharynx

Oral cavity	Oropharynx
Hard palate	Soft palate/uvula
Buccal mucosa right/left	Oropharyngeal sidewall right/left
Tongue (anterior 2/3)	The base of the tongue
Floor of mouth	Posterior pharyngeal wall

According to the 8th edition of the Union for International Cancer Control (UICC) tumor node metastasis (TNM) classification

Quality of life, local control, and OS 12 months after RT are the secondary endpoints of the trial. After 12 months of study specific follow-up, the patients will continue with regular clinical follow-up examinations. Also, patient-reported outcomes (PRO) and dental status, as well as plan comparisons and robustness analyzes, will be assessed as exploratory endpoints. Also, the functional and differentiation parameters, as well as the regenerative and radioprotective factors of gingival MSCs will be evaluated in vitro.

### Study visits and evaluation criteria

**Fig. 2:**
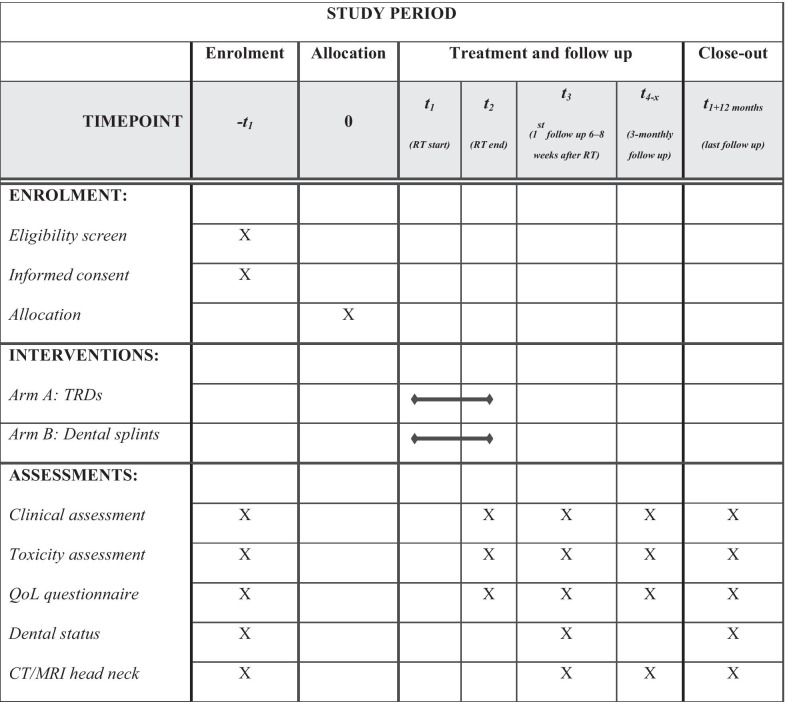
Schedule of enrolment, interventions, and assessments for the prospective randomized controlled GUARD trial (SPIRIT figure). Abbreviations: Radiotherapy (RT), tissue retraction devices (TRDs), quality of life (QoL), computed tomography (CT), magnetic resonance imaging (MRI)

The follow-up corresponds to the clinical routine, except for the quality of life questionnaires, PRO, and dental status assessment. The Quality of Life Questionnaire (QLQ) of the European Organization for Research and Treatment of Cancer (EORTC)—H&N35 [24], the Groningen Radiotherapy Induced Xerostomia questionnaire (GRIX) [25] and 12 HNC specific items from the CTC-PRO library [26] will be used in the current trial. The first study visit will be conducted 6 weeks after RT, and thereafter every 3 months within the first year after treatment. Detailed information on study visits and evaluation criteria is shown in Fig. 2.

Patients will be followed for at least 12 months after RT to document any acute and subacute toxicity from CTC v5.0 that is related to study treatment. Response to treatment and progression will be defined according to the most recent Response Evaluation Criteria in Solid Tumors (RECIST) 1.1. Patients with partial follow-up are weighted by the proportion of the follow-up time that is completed.

### Data management and statistics

The data is collected, managed, and processed electronically in the internal research database. The statistical analysis is based on the “Structure and Content of Clinical Study Reports” of the International Conference on Harmonization Guidelines and “Statistical Principles for Clinical Trials”.

#### Power calculation

The trial aims to demonstrate that the rate of oral mucositis in the experimental group, π_E_, is lower than the rate of oral mucositis in the control group, π_C_. Hence, the trial aims to reject the one-sided null hypothesis H_0_: π_E_ ≥ π_C_. We assumed that the rate of oral mucositis in certain anatomical regions in the experimental group will amount to π_E_ = 15%, while the rate of the control group is assumed to be π_E_ = 60%. The assumed rate in the control group is based on published data [4, 5, 27], while the rate in the experimental group reflects the assumption that the experimental intervention can achieve a decrease below grade III for the mucositis in the directly neighboring region of the tumor for 75% of all patients with mucositis. This hypothesis is based on preliminary clinical experience in ten patients treated with TRDs. Under these assumptions, 28 evaluable patients (14 per group) are required to achieve 80% power with a chi-square test at a one-sided 5% level of significance. Assuming a 15% dropout rate, 34 patients (17 per group) will be enrolled. The calculation of the sample size was performed using ADDPLAN v6.1.

#### Analysis of the primary endpoint

The null hypothesis π_E_ ≥ π_C_ will be evaluated at a one-sided significance level of 5% using a chi-square test. Also, the associated odds ratio for the treatment effect will be provided with a one-sided 95% confidence interval. The analysis will be performed based on the safety population, including all randomized patients who were treated according to planned therapy for at least one day. Missing values for the primary outcome will be replaced by multiple imputations using the fully conditional specification method [27]. Sensitivity analyzes will be performed via complete-case analysis and the best and worst-case analysis.

#### Analysis of secondary endpoints

Secondary endpoints for OS and local control will be assessed using Kaplan Meier estimators. 1-year survival rates and median times of events will be provided with 95% confidence intervals. Descriptive log-rank tests will be performed to compare the two treatment groups. All other secondary endpoints will be analyzed descriptively. The safety analysis will comprise a tabulation of absolute and relative frequencies for serious and adverse events, along with 95% confidence intervals.

The details of the statistical analysis will be defined in a statistical analysis plan (SAP) that will be finalized before the database lock. The analyzes will be performed with SAS v9.4 or higher.

### Ethics and safety considerations

The Ethics Committee of Heidelberg University (S-394/2020) approved the study protocol, patient information form, and informed consent statement. The clinical trial will be conducted following the latest version of the "Declaration of Helsinki". Regarding the performance, evaluation, and documentation of this study, Good Clinical Practice (GCP) recommendations have been taken into consideration. The regulations concerning medical confidentiality and data protection are fulfilled. Adverse events will be monitored and recorded following GCP guidelines. The biocompatibility and stability of the FREEPRINT® material used have already been approved for clinical application as a class IIa medical device. Therefore, a clinical investigation following the Medical Device Act does not apply to the current study.

## Discussion

The primary aim of the current phase II study is to evaluate the efficacy of TRDs for the radiation treatment of head and neck tumors. The distance between the tumor and normal tissue can be increased in certain anatomical regions through the use of individualized TRDs, thus protecting the healthy oral mucosa from unwanted radiation exposure. We hypothesize that TRDs can significantly reduce the rate of side effects, as reducing treatment margins by a few millimeters can significantly attenuate treatment toxicity [14]. This could even reduce clinically relevant complications (e.g. weight loss due to painful swallowing) and long-term sequelae (e.g. xerostomia). Also, the jaw spacing and protrusion of the lower jaw by TRDs make breathing easier during treatment sessions, with consequences for patient comfort. However, it is not clear which approach is the most appropriate to assess the tolerability of treatment. Recently, PROs have gained importance in comparison with the conventional, physician-reported toxicity assessments, and quality of life questionnaires [26]. Therefore, in the current trial, the GRIX questionnaire and 12 selected PRO-CTC items relevant to patients with head and neck tumors will be used, besides the physician-reported toxicity profile.

Besides the primary objective of the trial, the present study will investigate several additional physical-technical and clinical aspects of individualized 3D printed TRDs. We hypothesize that the robustness of RT plans concerning positional variability of bone and soft tissue structures could be improved by TRD-mediated fixation. The degree of dose reduction in the oral mucosa by the use of TRD is currently unknown and will be assessed by plan comparisons between both treatment groups and correlated with clinical findings. Comparisons of in silico intrapatient RT plans, using previously available diagnostic CT/MRI scans, could reveal more functional aspects of TRDs. We will elaborate on these findings for various treatment approaches, including IMRT and particle therapy, as part of the study.

Oral evaluation and care before RT are well established for patients with HNC [19]. However, the long-term consequences on the dental condition are most likely underestimated. The present study aims to contribute to the understanding and prevention of dental sequelae after RT through systematic evaluations by a specialist in prosthetic dentistry.

The exploratory objective of the trial is to investigate gingival MSCs, derived from clinically indicated dental extractions tissue. We anticipate that the regenerative and radioprotective properties of MSCs could further improve the clinical tolerability of RT. However, the indirect tumor protective effects of MSCs must be ruled out before clinical use.

## Data Availability

Not applicable.

## Abbreviations

CT: Computed tomography
CTC: Common Terminology Criteria
EORTC: European Organisation for Research and Treatment of Cancer
GCP: Good Clinical Practice
GRIX: Groningen Radiotherapy-Induced Xerostomia
HNC: Head and neck cancer
IMRT: Intensity-modulated radiotherapy
MRI: Magnetic resonance imaging
MSCs: Mesenchymal stem cells
PRO: Patient-reported outcomes
QLQ: Quality of Life Questionnaire
QUANTEC: Quantitative Analyses of Normal Tissue Effects in the Clinic
RIOM: Radiation-induced oral mucositis
RT: Radiotherapy
TRD: Tissue retraction device
